# Interleukin 17A Promotes Lymphocytes Adhesion and Induces CCL2 and CXCL1 Release from Brain Endothelial Cells

**DOI:** 10.3390/ijms18051000

**Published:** 2017-05-08

**Authors:** Dagmara Weronika Wojkowska, Piotr Szpakowski, Andrzej Glabinski

**Affiliations:** Department of Neurology and Stroke, Medical University of Lodz, Zeromskiego 113, 90-549 Lodz, Poland; piotr.szpakowski@umed.lodz.pl (P.S), andrzej.glabinski@umed.lodz.pl (A.G.)

**Keywords:** Th17 cells, blood–brain barrier, chemokines, VCAM-1, neuroinflammation, multiple sclerosis

## Abstract

The nature of the interaction between Th17 cells and the blood–brain barrier (BBB) is critical for the development of autoimmune inflammation in the central nervous system (CNS). Tumor necrosis factor alpha (TNF-α) or interleukin 17 (IL-17) stimulation is known to enhance the adherence of Th17 cells to the brain endothelium. The brain endothelial cells (bEnd.3) express Vascular cell adhesion molecule 1 (VCAM-1), the receptor responsible for inflammatory cell adhesion, which binds very late antigen 4 (VLA-4) on migrating effector lymphocytes at the early stage of brain inflammation. The present study examines the effect of the pro-inflammatory cytokines TNF-α and IL-17 on the adherence of Th17 cells to bEnd.3. The bEnd.3 cells were found to increase production of CCL2 and CXCL1 after stimulation by pro-inflammatory cytokines, while CCL2, CCL5, CCL20 and IL17 induced Th17 cell migration through a bEnd.3 monolayer. This observation may suggest potential therapeutic targets for the prevention of autoimmune neuroinflammation development in the CNS.

## 1. Introduction

Multiple sclerosis (MS) is a neurological disease wherein the myelin and neurons of the brain and spinal cord are destroyed by the immune system, a process believed to be mediated by CD4^+^ T cells [1,2]. Demyelination of neurons leads to impaired communication within the central nervous system (CNS). In consequence, patients suffering from MS have several neurological signs and symptoms including muscle weakness, lack of coordination of movements and sensory disturbances [3]. Although the etiology of this disease remains unknown and no satisfactory therapy exists, several studies have shown that Th17 cells are important components in the mechanism of MS pathogenesis [4,5]. Th17 cells produce a range of inflammatory cytokines, including interleukin 17 A/F (IL-17A/F), interleukin 21 (IL-21) and interleukin 22 (IL-22), which are involved in the development of inflammatory foci in the CNS, and neutralization of the key cytokine IL-17 ameliorated symptoms in an animal model of MS: experimental autoimmune encephalomyelitis (EAE). The critical step of this process is transmigration of Th17 cells through the blood–brain barrier (BBB) [6,7].

The BBB is a physical barrier between the blood and the CNS, which protects the CNS from harmful factors contained in blood and elements of the immune system, and provides homeostasis in the brain environment. It is mostly composed of brain endothelial cells tightly connected by specific protein complexes (tight junctions). These cells have a high level of metabolic activity that enables selective delivery of substances from the blood to the CNS [8]. Brain endothelial cells express a number of adhesion molecules, including intercellular and vascular adhesion molecules such as vascular cell adhesion molecule 1 (VCAM-1), and P- and E-selectin, and release several inflammatory mediators [9,10]. Interestingly, the tight junctions of the BBB are destabilized when IL-17 and IL-22 bind to their receptors expressed on the BBB cells [7]. This deregulation increases the permeability of the BBB to leukocytes, which is considered to be the first step of MS development [11].

The migration of inflammatory cells to the CNS is a multistep process and involves various adhesion molecules. It has been shown that VCAM-1 mediates the adhesion of mononuclear cells to the vascular endothelium [12]. VCAM-1 interactions with its ligand Very Late Antigen 4 (VLA-4) on lymphocytes mediates processes of cell rolling on the endothelial cells surface, as also are necessary to lymphocyte stoppage and attachment. Recent studies have shown that Th17 cells also express VLA-4, and that this receptor is important in the development of MS [13,14,15]. Molecules mediating lymphocyte–endothelium interactions are the aim of MS therapies. Natalizumab is the first monoclonal antibody available in MS therapy which inhibits interactions between VLA-4 on T cells and VCAM-1 on the brain endothelium [16]. This drug is very effective in MS therapy but due to its side effects like PML (progressive multifocal leukoencephalopathy), liver dysfunction, and many others, it is still necessary to develop new, safer and more effective therapies for MS [17,18].

Recent findings suggest that the immune surveillance of the brain occurs, and, under physiological conditions, the antigen presenting cells as well as T-lymphocytes may migrate through the endothelium and accumulate in perivascular spaces of BBB without crossing the glia lamina. In these spaces the brain antigens presentation to cognate peripherally activated lymphocytes may occur. In vitro and in situ studies have shown that, at the onset of MS, peripherally activated Th17 cells may reach the perivascular space and secrete IL-17 after recognition of cognate antigens presented by antigen presenting cells [19]. This suggests that the presence of Th17 cells in the perivascular area may affect BBB permeability and promote leucocytes migration [19,20]. Inflammatory cell migration through the BBB depends not only on the adhesion receptors on the surface of the brain endothelium, but also on their attraction by such chemotactic cytokines as CCL2, CCL5 and CCL20 [21].

The aim of this study was to examine the interactions between Th17 cells and brain endothelium in vitro with regard to their influence on the pathogenesis of MS.

## 2. Results

### 2.1. Th17 Cell Adherence to Brain Endothelium

**Figure 1:**
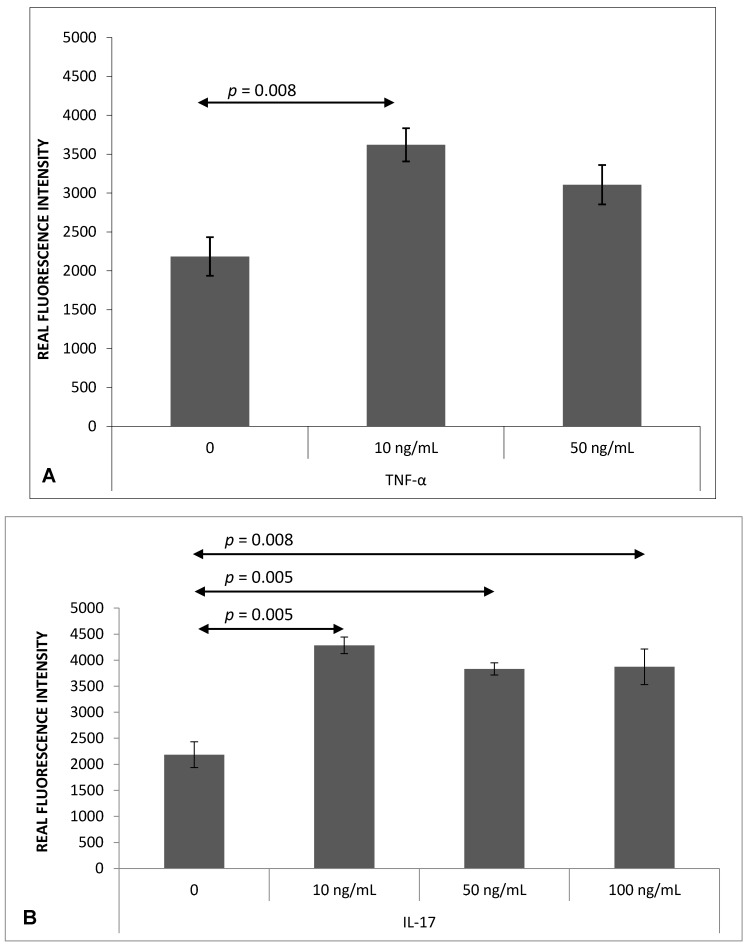
Adherence of mouse Th17 cells to murine brain endothelium bEnd.3 after 4 h of stimulation with TNF-α (**A**) or IL-17 (**B**). Adherence was measured as described in Materials and Methods. Data shown as mean ± SEM; *n* = 6 for the numbers of performed experiments.

Significantly greater Th17 cells adherence to bEnd.3 cells was observed after stimulation with TNF-*α* (10 ng/mL, *p* = 0.008) (Figure 1A) or IL-17 (10 ng/mL, *p* = 0.005; 50 ng/mL, *p* = 0.005 and 100 ng/mL, *p* = 0.008; respectively) (Figure 1B).

### 2.2. Expression of VCAM-1 Receptor on Brain Endothelium

**Figure 2:**
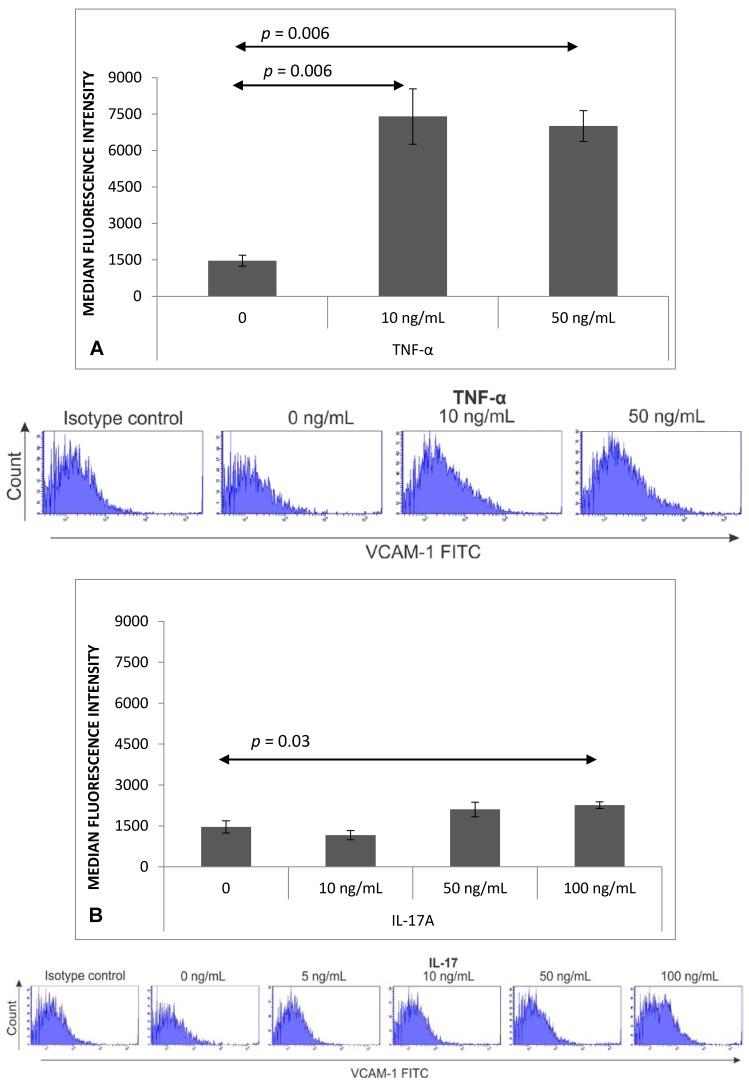
VCAM-1 expression on mouse brain endothelial cell line (bEnd.3) after 4 h of stimulation with TNF-α (**A**) or IL-17 (**B**). Expression was measured as described in Materials and Methods. Results shown as mean ± SEM and representative flow cytometry histograms; *n* = 5 for TNF-α stimulation and *n* = 6 for IL-17 stimulation and unstimulated control.

Significantly greater VCAM-1 expression was observed in brain endothelial cells after stimulation with TNF-α (10 ng/mL, *p* = 0.006 and 50 ng/mL, *p* = 0.006) (Figure 2A). Expression of VCAM-1 was also upregulated on bEnd.3 cells stimulated with IL-17 (100 ng/mL; *p* = 0.03) (Figure 2B).

### 2.3. Chemokine Production by Brain Endothelium

**Figure 3:**
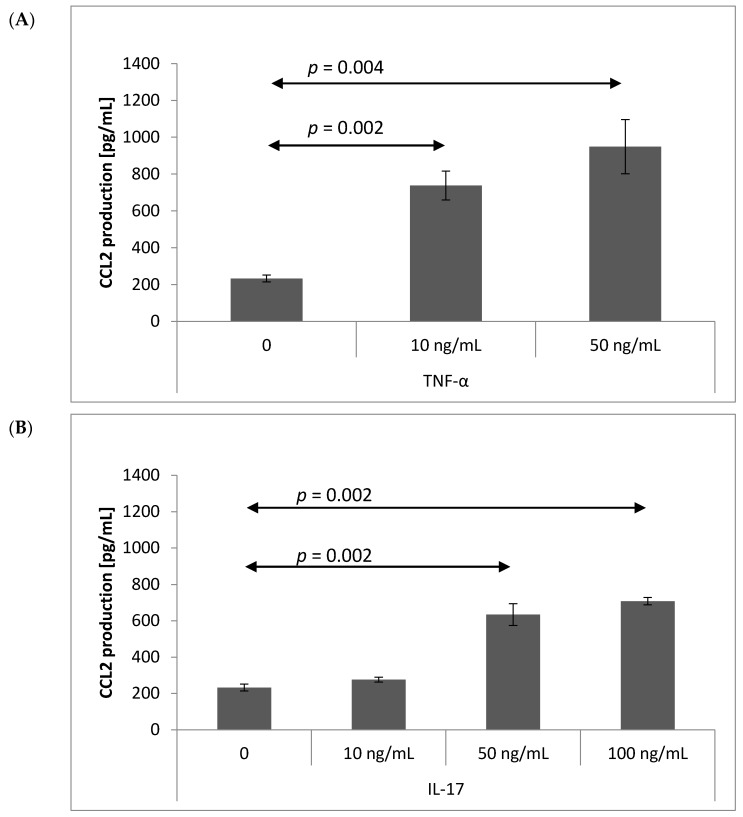

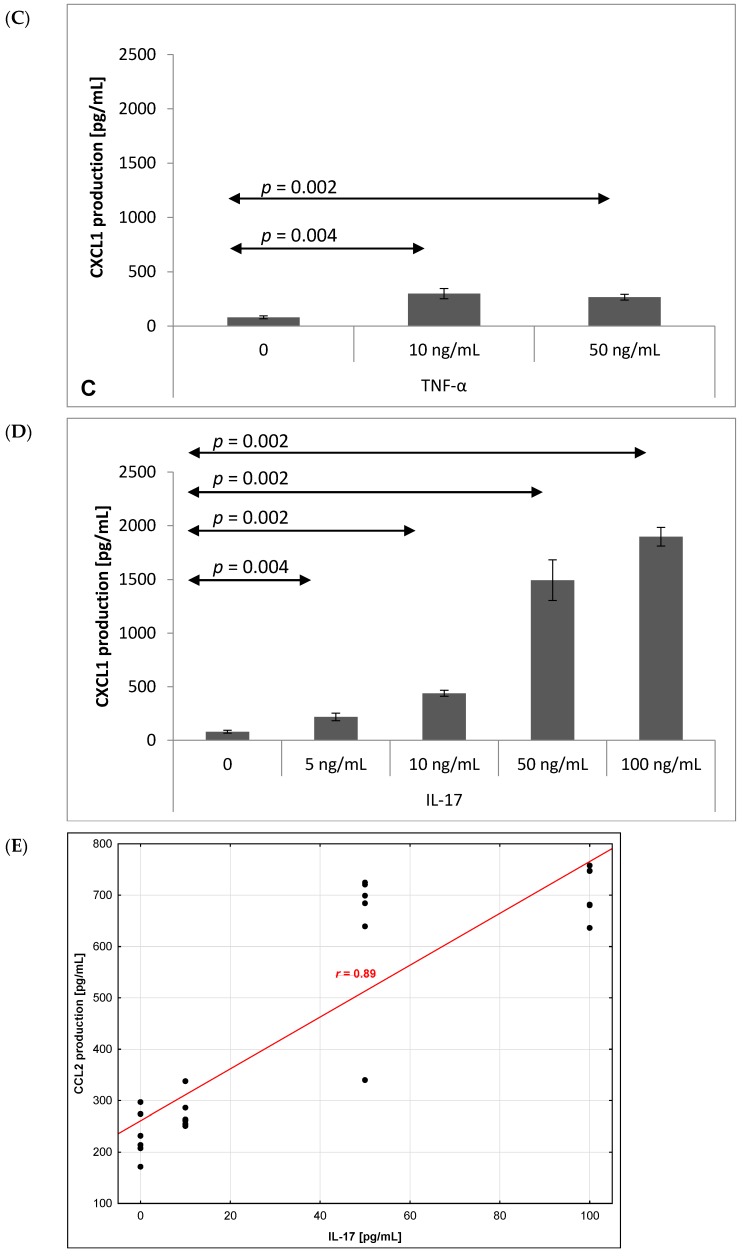

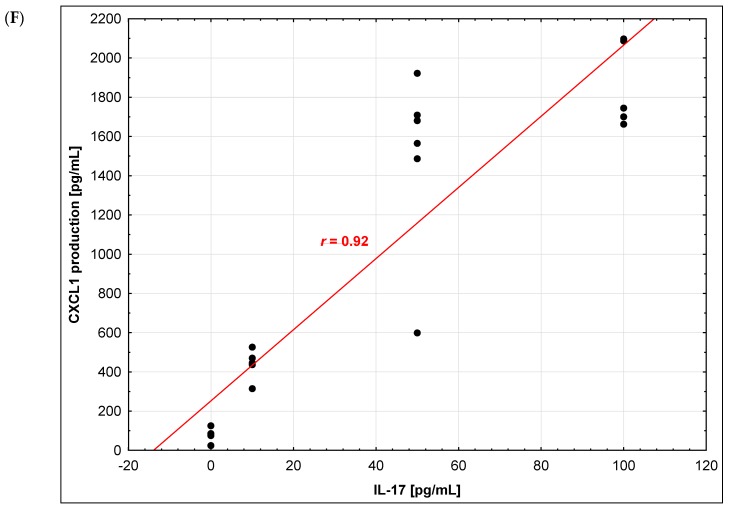
CCL2 (**A**,**B**) and CXCL1 (**C**,**D**) chemokines production by mouse brain endothelial cell line (bEnd.3) after 4 h of stimulation with TNF-α (**A**,**C**) or IL-17 (**B**,**D**). Production was measured as described in Materials and Methods. Data expressed as mean ± SEM. Correlation between IL-17 stimulation and production of CCL2 (**E**) or CXCL1 (**F**) by brain endothelium. Underlined *r* values are considered statistically significant (Pearson’s test, *p* < 0.05); *n* = 6 for the numbers of performed experiments.

CCL2 production was significantly increased in brain endothelial cells after TNF-α stimulation (10 ng/mL, *p* = 0.002 and 50 ng/mL, *p* = 0.004) (Figure 3A). Higher concentrations of IL-17 also significantly increased CCL2 production by brain endothelial cells (50 ng/mL, *p* = 0.002 and 100 ng/mL, *p* = 0.002) (Figure 3B). Similarly, the production of chemokine CXCL1 by bEnd.3 was significantly upregulated by TNF-α (10 ng/mL, *p* = 0.004 and 50 ng/mL, *p* = 0.002) (Figure 3C) and IL-17 (5 ng/mL, *p* = 0.004 and 10, 50, 100 ng/mL, all *p* = 0.002) (Figure 3D). Dose-dependent production of CCL2 (Figure 3E) and CXCL1 (Figure 3F) was observed in brain endothelial cells following IL-17 induction (Pearson correlation coefficient *r* = 0.88, *p* < 0.001). The chemokines CCL5 and CCL20 were not produced by bEnd.3 cells in response to TNF-α or IL-17 stimulation (data not shown).

### 2.4. Chemokine-Induced Transmigration of Th17 Cells through the Brain Endothelium

**Figure 4:**
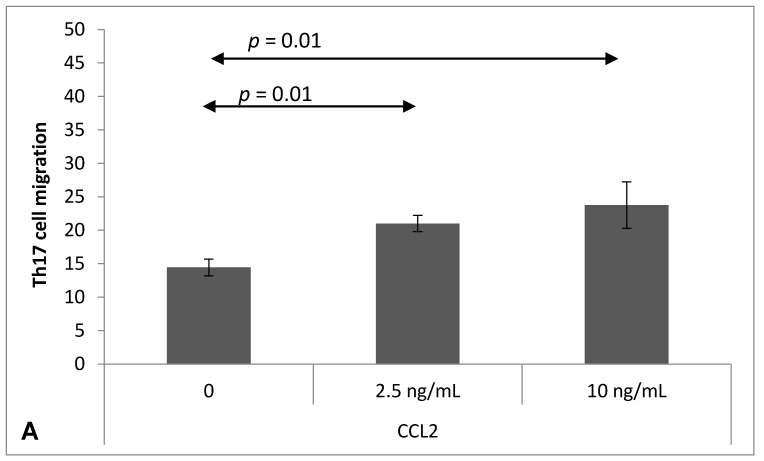

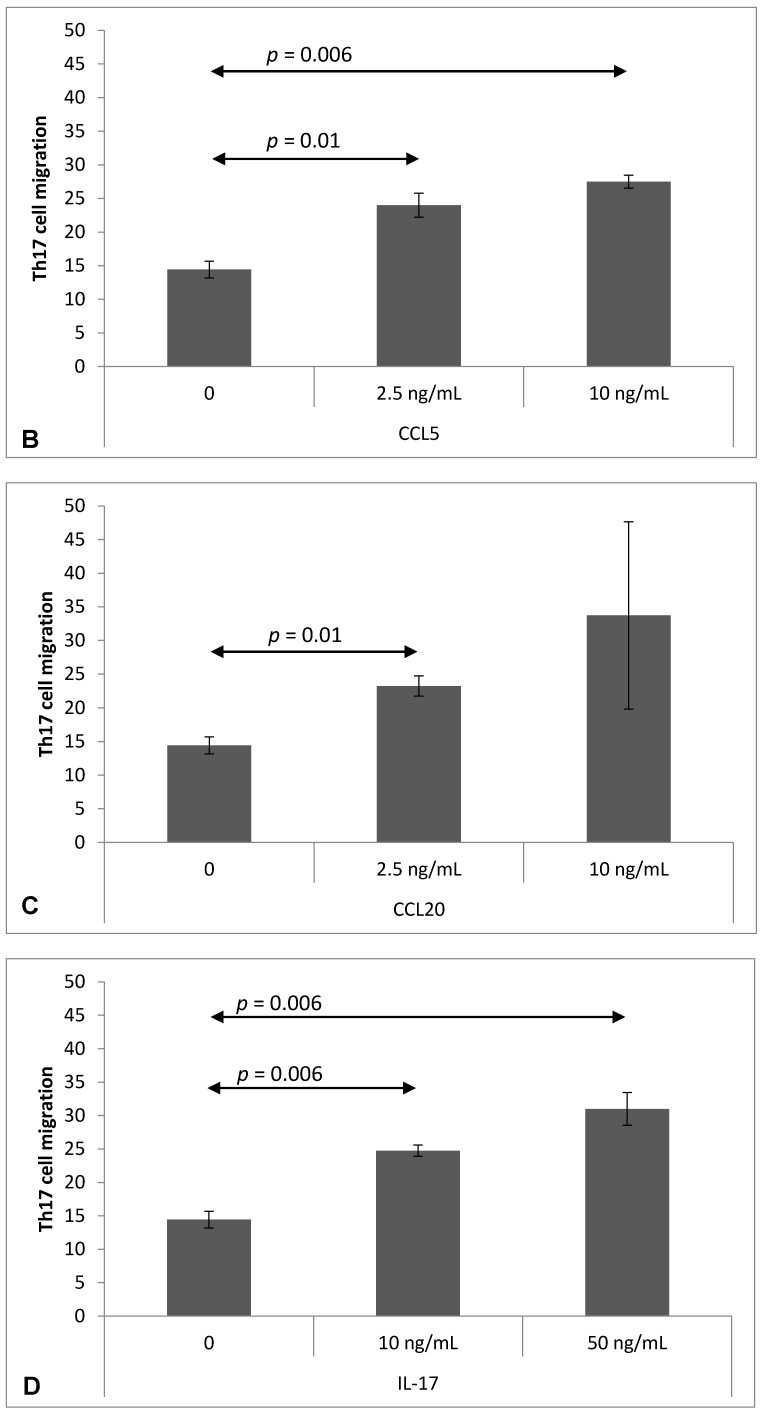
Th17 cells migration through mouse brain endothelium (bEnd.3), stimulated by CCL2 (**A**); CCL5 (**B**); CCL20 (**C**) or IL-17 (**D**). Migration was measured as described in Materials and Methods. Data expressed as mean ± SEM; *n* = 3 for the numbers of performed experiments

Chemokines CCL2 (2.5 ng/mL, *p* = 0.01; 10 ng/mL, *p* = 0.01) (Figure 4A), CCL5 (2.5 ng/mL, *p* = 0.01; 10 ng/mL, *p* = 0.006) (Figure 4B), CCL20 (2.5 ng/mL, *p* = 0.01) (Figure 4C) or IL-17 (10 ng/mL, *p* = 0.006; 50 ng/mL, *p* = 0.006) (Figure 4D) significantly stimulated Th17 cell transmigration through the bEnd.3 monolayer.

## 3. Discussion

A wealth of evidence indicates that Th17 cells, a recently-described subpopulation of lymphocytes important for induction and maintaining of immune response, play a pivotal role in the development of autoimmunity. Our results suggest that IL-17, a key product of activated Th17 cells, is able to modify the functions of brain endothelial cells: the most important compartment of BBB. IL-17 may dramatically increase the adhesion of Th17 cells to the brain endothelium, thus initiating their migration to the brain parenchyma. Our findings indicate that Th17 cells strongly adhere to brain endothelial cells following TNF-α stimulation. Mardiguian et al. report that prophylactic administration of anti-IL17A downregulates the expression of VCAM-1 on endothelial cells, resulting in reduced clinical disability in EAE mice; the administration of anti-IL17A after the appearance of symptoms prolonged remission and ameliorated the symptoms [22].

Surprisingly, the observed IL-17-dependent adhesion of Th17 cells to the brain endothelium was not found to be associated with VCAM-1 expression. A significant increase in VCAM-1 expression was observed only for cells stimulated with the highest concentration of IL-17 (100 ng/mL). TNF-α is strong inducer of leucocyte diapedesis; its role for induction of VCAM-1 expression on bEnd.3 endothelial cells was described by Sikorski et al. [10]. Interestingly, endothelial cells stimulated with TNF-α, which is overproduced during inflammation in MS [23], resulted in much higher VCAM-1 expression than IL-17 stimulation, while increased VCAM-1 expression was also noted following stimulation with a combination of TNF-α and the lowest concentration of IL-17 [24]. It has also been reported that endothelium activated by IL-17 and TNF-α synergistically enhanced leukocyte rolling, which influenced the gathering of pro-inflammatory cells around the inflamed area [25].

Our results suggest that stimulated bEnd.3 cells are able to express the VCAM-1 molecules responsible for effector lymphocyte adhesion; however, the IL-17-dependent Th17 adhesion to bEnd.3 cells may not be associated with changes in VCAM-1 expression. This may be explained by the fact that lymphocyte rolling is only possible when specific receptors—including selectins localized on the endothelium—interact with their ligands on the Th17 cells. In later experiments, Th17 cells were observed to transmigrate through the brain endothelium monolayer in response to stimulation by IL-17 levels too low to increase VCAM-1 expression; however, these levels were sufficient for adhesion induction, which suggests that other adhesion molecules may play a role in the process. Interestingly, Huppert et al. [6] report an increase in ICAM-1 expression, but no changes in VCAM-1, E-selectin or P-selectin expression on endothelium cells in response to IL-17 application; however, their study only examined endothelium cells stimulated with 100 ng/mL IL-17 for a shorter period, i.e. three hours. In contrast, the present study also examines the impact of lower doses of IL-17 on endothelium–Th17 cell interactions.

Although several studies have shown that a large number of chemokines can be produced in the CNS, our present findings indicate that stimulated brain endothelial cells produce chemokines CCL2 and CXCL1, but not CCL5 or CCL20. IL-17 chiefly acts by inducing pro-inflammatory and mononuclear cell cytokine secretion, thus opening the gates for neutrophil and T lymphocyte entry and propagating the immune response. Our results show that IL-17 acts directly on the endothelial cells, inducing the release of CCL2 and CXCL1 chemokines in a dose-dependent manner, which may be an important feature of Th17–BBB interactions. In addition, the influence of IL-17 on the endothelium appears to be related to cytokine concentration, and so may promote different aspects of the immune response: lower concentrations of IL-17 promote the secretion of CXCL1 rather than CCL2, whereas high doses of IL-17 induce both CXCL1 and CCL2.

CCL2, formerly known as monocyte chemoattractant protein 1 (MCP-1), is a small cytokine belonging to the CC chemokine family with a molecular weight of approximately 13 kDa. CCL2 is widely considered to be a critical mediator of inflammation localized in the central and periphery nervous systems [26,27,28]. Elevated levels of CCL2 have been reported by numerous authors in EAE in the brain [29,30,31,32], and neurons such as astrocytes and microglia cells are also known to be potential producers of CCL2 in the CNS. CCL2 attracts mainly T-cells, but also monocytes, dendritic cells, and basophils to the inflamed areas, and is undetectable in the healthy brain. It has been proposed that CCL2 allows astrocytes to regulate the permeability of the BBB and influence the integrity of endothelial cell culture by the reorganization of tight junction proteins [33,34,35]. Recent analyses of cell selective knockouts based on 3D confocal microscopy suggest that CCL2 produced by the endothelium plays a role in the initiation of adhesion to the endothelium and the regulation of post-adhesion leukocyte trafficking across the endothelial layer, while astrocytes producing CCL2 may mediate leukocyte penetration in the parenchyma [28]. Our findings suggest that Th17 cells producing IL-17 may stimulate the release of CCL2 from endothelial cells, resulting in BBB disruption in a positive feedback loop. The results of the chemotactic assay revealed intensified Th17 cell migration through the endothelial layer following induction by CCL2, CCL5 and CCL20; although these are known chemoattractants for T lymphocytes, the same was also observed for IL-17, which does not possess any such activity. However, as shown previously, IL-17 may also induce the release of chemokines CCL2 and CXCL1 from endothelial cells.

CXCL1 belongs to the family of CXC chemokines, which are known for their ability to attract neutrophils. IL-17 has been found to stabilize mRNA for CXCL1, thus increasing its half-life [36]. A number of studies have demonstrated CXCL1 secretion from epithelial and mesodermal cells, but little is known about its production by the endothelium. Recently, Makito et al. described CXCL1 production in an autocrine manner by human endothelial cells and its role in angiogenesis [37]. CXCL1 production is also known to be enhanced by inflammatory cytokines such as TNF-α and IL-1β in human endothelial cells [38].

Our previous work on EAE indicated that CXCR2-positive Th17 cells predominate in the CNS during the preclinical phase and Th17 in the symptomatic phase, and that a significant increase in neutrophil accumulation occurs during the preclinical phase [39], which may suggest that CXCL1 plays a role in both neutrophil regulation and Th17 cell attraction into the brain during the development of EAE. The ability of Th17 cells to induce CXCL1 production via IL-17 secretion has been postulated as a pathogenic pathway linking Th17 cells and neutrophils via endothelial secretion of ELR CXC chemokines. It is possible that IL-17 favors CXCL1 production in the brain endothelium in the early stages of MS and EAE, thus attracting neutrophils, inducing them to secrete pro-inflammatory cytokines including IL-1, TNF-α and IL-8, which subsequently provide entry for lymphocytes and monocytes [40]. In this case, the Th17 cells serve to provide entry to the CNS for neutrophils. Increasing IL-17 production stimulates greater CCL2 production, leading to the attraction of further inflammatory cells to the brain.

The chemotaxis assay found that both CCL2 and CCL5 were able to attract Th17 cells, and that these cells can easily cross the bEnd.3 layer which acts as the BBB in the CCL2 environment. CCL5 is expressed by various cell types; it acts as a chemoattractant for T cells and monocytes, increases the adherence of monocytes to endothelial cells, and plays an active role in recruiting leukocytes to inflammatory sites. The application of anti-CCL5 antibodies can dramatically inhibit this process [41].

Another important chemokine in MS pathogenesis is CCL20, which may attract T and B cells and dendritic cells [42]. It has been proven that human Th17 cells with the CCR6 receptor can express 100 times more IL-17 than those without [43]. CCL20 plays an important role in the migration of Th17 cells to inflammatory sites and can also induce IL-17 production [44]. Following IL-6 and TGF-α application, Th17 cells are capable of expressing CCL20, which is an interesting mechanism of enhancing the Th17-dependent immune response [44]. Our findings demonstrate that either spontaneous chemotaxis of Th17 cells, or their migration through the bEnd.3 cell towards CCL20, was similar and concentration-dependent. The expression of CCL20 could represent a gateway by which CCR6 leukocytes can cross the BBB and reach CNS parenchyma even without the presence of pathogenic T cells.

In summary, migration of Th17 cells through the brain endothelium is a complicated process requiring expression of several inflammatory mediators. Our data suggest that, during this process, expression of VCAM-1 is upregulated on the brain endothelium. Moreover, chemokines CCL2 and CXCL1 are produced by brain endothelium after stimulation with inflammatory cytokines TNF-α and IL17. Targeting those processes could be a promising mechanism of future MS therapies.

## 4. Materials and Methods

### 4.1. Mice and Tissue Collection

All experiments were carried on female SJL (Swiss Jim Lambert) mice aged 8 to 12 weeks purchased from The Jackson Laboratory (Bar Harbor, ME, USA). The mice were housed under standard conditions at the Animal House of the Medical University of Lodz, Lodz, Poland. All animal procedures and protocols were conducted in accordance with the Guide for the Care and Use of Laboratory Animals of the National Animal Care Committee and were approved by Local Ethical Committee for Animal Research in Lodz (identification code: 46/LB 563/2011) on 18 July 2011. All efforts were made to minimize animal suffering. The spleens were collected from mice anesthetized intraperitoneally with ketamine and xylazine solution (0.1 mL/20 g mouse). The mice were perfused transcardially with cold phosphate buffered saline (PBS) followed by PBS containing heparin.

### 4.2. Isolation of CD4^+^ T Cells and Th17 Cell Culture

The spleens were rubbed through a nylon sieve (70 μm, BD Falcon, Corning, NY, USA) and washed with PBS. The resulting cell suspensions were centrifuged for 10 min at 350× *g* (4 °C). The splenocytes were resuspended in 5 mL of red blood cell lysis buffer and kept for 5 min on ice before being washed with PBS and centrifuged at 350× *g* for 10 min at 4 °C. To obtain mouse CD4^+^ cells, magnetic separation was performed using a CD4^+^ T Cell Isolation Kit (Miltenyi Biotec, Bergisch Gladbach, Germany), according to the manufacturer’s protocol. Cell number and viability were determined using trypan blue (Sigma-Aldrich, USA) staining and counting in a Bürker chamber (Sigma-Aldrich, St. Louis, MO, USA) under a light microscope. Th17 cells were obtained from collected CD4^+^ T cells using the Flow Cellect^™^ Mouse Th17 Differentiation Tool Kit (Millipore Merck, Darmstadt, Germany) according to the manufacturer’s protocol. Detailed assay instructions certify to gain complete Th17 cell generation in 6 days. After seeding 2 × 10^5^ CD4^+^ cells on day 1 into each well of the kit’s plate, on day 6 the total number of differentiated cells were approximately 1–5 × 10^6^. All cell types were stained with trypan blue solution and counted in a Bürker chamber under a light microscope.

### 4.3. Mouse Brain Endothelial Cell (MBEC) Culture

Murine bEnd.3 cells (ATCC^®^ CRL-2299) were purchased from the cell culture collections of ATCC-LGC Standards (Manassas, VA, USA). The cells were maintained in Dulbecco’s modified Eagle’s medium (DMEM, ATCC-LGC Standards) with 10% fetal bovine serum (FBS, ATCC-LGC Standards) and cultured in tissue culture flasks to 80% confluence.

### 4.4. Analysis of Th17 Cell Adherence to MBEC

The cell adhesion assay was performed with the use of the CytoSelect™ Leukocyte-Endothelium Adhesion Assay (Cell Biolabs Inc, San Diego, CA, USA) according to the manufacturer’s protocol. Briefly, bEnd.3 cells were cultured to 80% confluence on gelatin-coated 96-well culture plates (Greiner, Olbendorf, Austria) in DMEM culture medium enriched with 10% FBS. Following this, the culture medium was removed and the wells washed with DMEM medium. Fresh culture medium with various concentrations of TNF-α (10 and 50 ng/mL) or IL-17 (5, 10, 50 and 100 ng/mL) was then added to the wells, with culture medium alone being used for negative controls.

After a four-hour stimulation period, the culture medium was removed and cells were washed twice with DMEM medium. The Th17 cells were labeled with fluorescent dye (LeukoTracker, Cell Biolabs Inc., San Diego, CA, USA), and 8 × 10^4^ of labeled cells in DMEM medium were added to each well. After one hour, the culture medium with non-adherent cells was removed, and the remaining cells were washed three times with the wash buffer solution, and lysed with lysing buffer. Fluorescence was determined on a Victor 2 (Perkin Elmer, Waltham, MA, USA) fluorescence reader at 480/520 nm.

### 4.5. Measurement of Chemokine Level by ELISA

After culturing for four hours, the culture medium was collected from the bEnd.3 cells, and the medium with cytokines after stimulation and medium from control cells were centrifuged at 400× *g* for 10 min at 20 °C, aliquoted and stored at −80 °C for further ELISA measurements. Each culture was performed as six replicates. The CCL5, CCL20 and CXCL1 levels were measured using DuoSet ELISA (R&D Systems, Inc., Minneapolis, MN, USA), while CCL2 levels were assessed with Ready-Set-Go ELISA (eBioscience, San Diego, CA, USA). All measurements were carried out according to the manufacturer’s protocols.

### 4.6. Flow Cytometry Analysis of VCAM-1 Expression on MBEC

The MBEC cultures were grown in whole culture medium on a 24-well culture plate until 80% confluence. The medium was then replaced with fresh culture medium containing 10 or 50 ng/mL TNF-α (eBioscience, San Diego, CA, USA), or 5, 10, 50 or 100 ng/mL IL-17 (eBioscience). Control wells were set up consisting of bEnd.3 cells in culture medium alone.

The cells were stimulated for 3 h at 37 °C in 5% CO_2_. After stimulation, the medium was collected and stored for further ELISA measurements. The cells were washed with PBS and treated with trypsin to detach them from the plate surface. The single-cell suspensions (10^6^ cells) were labeled with FITC-conjugated antibody directed against VCAM-1 (rat anti-mouse CD106 monoclonal antibody, clone 429, cat no. 105705, BioLegend, San Diego, CA, USA). Flow cytometry was performed using a BD LSR II flow cytometer (Becton Dickinson, Franklin Lakes, NJ, USA) and analyzed with BD FACSDIVA 6.0 software. Isotype-matched rat antibodies were used as negative controls for staining.

### 4.7. Analysis of Th17 Cell Migration through the Brain Endothelium

Chemotaxis of Th17 cells across bEnd.3 layer was tested in vitro using Transwell culture inserts with 5 μm pores. First, 1 × 10^5^ of bEnd.3 cells were seeded into inserts and incubated for 24 h at 37 °C, 5% CO_2_. After incubation, confluence on the inserts was checked in light microscope and when the whole insert was covered with monolayer of bEnd.3 cells, the medium was gently removed and different concentrations of chemokines were added to the plate wells: CCL2 (2.5, 10 ng/mL), CCL5 (2.5, 10 ng/mL), CCL20 (2.5, 10 ng/mL) and IL-17 (10, 50 ng/mL). All concentrations of chemokines were prepared in culture medium and 700 μL volumes were added to each well of a 24-well plate, with each concentration added in duplicate. The control cultures were grown in medium alone.

Following this, 100 μL of culture medium with 4 × 10^5^ Th17 cells was added to each Transwell insert, and the plates were incubated for three hours at 37 °C, 5% CO_2_. After incubation, the migrated cells were harvested from the lower compartment of the chemotaxis chamber, stained with trypan blue solution and counted in the Bürker chamber under a light microscope. Results are reported as the number of cells that migrated to the medium with chemokine to the number of cells that migrated to medium alone.

### 4.8. Statistical Analysis

For statistical analyses, the non-parametric *U* Mann−Whitney test and Pearson correlation test were used. The normality of variables distribution was checked with the Kolmogorov−Smirnov test. All statistical calculations were performed with Statistica 12 statistical analysis software (StatSoft Inc., Tulsa, OK, USA). A value of *p* < 0.05 was considered statistically significant. Data was shown as mean ± SEM.
